# Assessing the Visualization-Based Decision Support System for Environmental Impact Assessments

**DOI:** 10.3390/ijerph19031345

**Published:** 2022-01-25

**Authors:** Seo-young Lee, Sanghee Shin, Hakjoon Kim, Min-Kyung Kim, So-Yeon Yoon, Sangdon Lee

**Affiliations:** 1 College of Human Ecology, Cornell University, Ithaca, NY 14853, USA; sl2668@cornell.edu (S.-y.L.); sy492@cornell.edu (S.-Y.Y.); 2 Gaia 3D Incorporation, Seoul 08611, Korea; shshin@gaia3d.com (S.S.); hjkim@gaia3d.com (H.K.); 3 College of Engineering, Ewha Womans University, Seoul 03760, Korea; enviecol@ewha.ac.kr

**Keywords:** graphics, sustainability, environmental literacy, Likert scale, usability test

## Abstract

Even though environmental impact assessments (EIAs) have been an important tool for environmental decision-making, most EIAs are published as a mix of text and tabular data that is not easily accessible to or understandable for the public. In this paper, we present a decision support system (DSS) that supports the decision-making of stakeholders in the EIA stage. The system was designed to improve the public’s understanding of stakeholders before and after a construction project by providing visualization of key environmental elements. We recruited 107 participants to test the usability of the system and examined the impacts of individual differences between the participants on their perceptions of the system, including their environmental expertise and computer self-efficacy. The results showed that the proposed system had high usability, especially for users with high computational efficacy and environment expertise. The system could thus help to improve the communication between the public and experts during public hearings and enhance the environmental literacy of the public.

## 1. Introduction

An environmental impact assessment (EIA) is a decision-making tool that helps to evaluate the likely positive and negative environmental impacts of a proposed project or development while also considering interrelated socio-economic, cultural, and health effects [1,2]. An EIA is designed to review, predict, and assess the potential environmental impacts of a project before its plan is finalized so that strategies can be developed to prevent or minimize possible adverse effects on the environment [2,3,4]. Although the EIA process may vary around the world, it is important to determine whether the predicted impacts occurred and whether the proposed mitigation measures were implemented. In particular, the early stages of an EIA in the process of public hearing/comments could lead to more engagement between the affected community and the developers [2,5].

In addition, in the upper level of the EIA process, SEA (strategic environmental assessment), the usability of visualization presumably could help SEA practitioners and planners in terms of more environmentally friendly development, a smoother planning process, greater plan transparency, and the like [6]. The visualization should bring public involvement of the EIA and SEA to speed up plans, policies, and projects by reducing the environmental impacts and improving the environmental benefits of plans, acting as an instrument of knowledge brokerage, and improve public participation in both levels [7].

There are some limitations that can hinder the communication between stakeholders in the process of environment-related decision-making [8]. In an EIA as well as SEA, experts often use only text and tables with numerical figures to present their findings to the public. Many stakeholders, such as the public, developers, administrators, and engineers, are involved in the decision-making process, and the role of the public and their participation is especially important. However, because the general public lacks professional knowledge regarding the environment, it is difficult for them to fully understand the details of projects under consideration. This lack of understanding could lead to serious miscommunication between stakeholders, inefficient decision-making, and unintended project outcomes. Previous studies only demonstrated modelled using a Bayesian network (BN) [9] and ANP (analytical network process) [10], but this lacks limited access to the internet (w/o installation of software). Furthermore, our study used real-time movement of water, airflow, and oil spill so that a modification of inputs can allow users to test alternatives in different ways, and this is a part of a wholistic model with visualization for a decision-making process.

To resolve this problem, a tool that can allow the public to easily understand the post-development associated with the EIA process is needed. Using this tool during the public review stage of a project, concerned citizens would be better able to understand the potential environmental consequences of a construction project. In this respect, the use of suitable data visualization elements such as maps, charts, and simulations would be useful because they can enhance the delivery of information for environmental topics [4]. It is also important for the tool to be accessible, which would encourage more widespread use and promote effective decision-making. Data visualization can also be important when conveying information about health impacts. For example, it has been found that how an impact assessment on the environmental and health impacts of coal mining is implemented and presented affects the outcomes of the project [11].

The main purpose of this paper is to design a system that provides effective data visualizations for environmental information that can be used to improve the communication between stakeholders. We thus propose a decision support system (DSS) that visualizes the environmental conditions before and after a proposed construction project to help EIA stakeholders communicate freely and make reasonable decisions. System usability testing that examined user satisfaction with the system was conducted, with the results analyzed depending on the individual differences of the respondents, in particular their computer self-efficacy and environmental expertise.

In the remainder of the paper, we first review past studies on EIAs and DSSs. We then describe the design and development of the system, before presenting the findings of the system usability testing. A discussion of these results and suggestions for future research then conclude the paper.

## 2. Related Works

### 2.1. Environmental Impact Assessment (EIA)

An EIA is a decision-making process that is used to predict the effects of a proposed construction or development project. An EIA is typically a detailed report that accompanies proposals for legislation and other major federal actions that significantly affect the quality of the human environment [12]. In the United States, EIAs obtained formal status in 1969 with the enactment of the National Environmental Policy Act (NEPA) [3].

There are three major stakeholders in an EIA: the general public, engineers/developers, and administrators. The public includes residents living in the residential development area, while the developers are those involved in developing the area, including the Water Resources Corporation (WRC), the Express Highway Corporation, and housing developers. Engineers are experts hired by developers to submit an environmental impact statement (EIS) because developers usually lack sufficient environmental expertise. Administrators include government officials who should produce records of decision (RoDs). A DSS would be most beneficial to the general public and administrators, particularly during the modification of a development plan. Developing a visualization-based DSS would be of most use for the general public to increase their understanding of and participation in EIAs.

**Figure 1:**
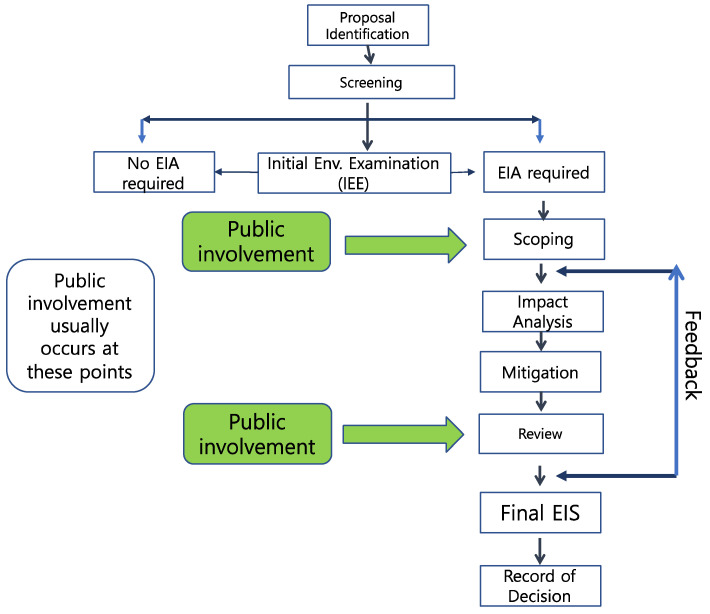
General process of EIA and stages with public involvement based on the environmental impact assessment.

Figure 1 presents the EIA process and stages that include public involvement before the final EIS is released (Figure 1), and in the upper level the public involvement should occur twice in the SEA. This process is also presented in Section 102 of NEPA [3]. The arrows on the right side indicate the feedback from the reviewers on the EIA if problems arise. The scoping process can be more detailed depending on the needs of the public and other stakeholders. Generally, an EIA is categorized into six environments in South Korea (Table 1).

### 2.2. Decision Support System (DSS)

A DSS is a computer-based interactive system that supports the decision process by providing current, timely, and manipulable information [4,13,14]. It supports decision-makers rather than replacing them using data and models. DSSs help to solve problems that consist of a diverse range of structured and unstructured tasks and have mostly been employed in the design and engineering fields [1,4,15,16,17].

Several studies have focused on the use of environmental DSSs for ecology and sustainability purposes [18,19,20]. These DSSs have been used to organize and manage information [20] and some have been developed using spatial data from a GIS database [19]. An increasing number of studies have employed advanced machine learning techniques in environmental DSSs. One study introduced the engineering machine-learning automation platform (EMAP), which supports the analysis of big data in the initial stages of the decision-making process, thus helping the contractors of plant projects to make better decisions in response to large amounts of complicated data [21,22].

To the best of our knowledge, few studies have adopted a DSS to support the EIA process, especially to improve the communication between stakeholders. The present study thus specifically focused on developing a DSS for an EIA that visually presents the potential changes to the environment due to a construction project based on spatial and time data. This is because data visualizations can increase the readability of environment-related information [4]. By employing the developed DSS in the EIA process, decision-making can be supported. The purpose of the DSS in the present study differs slightly from that of previous systems [21,22]. It does not focus solely on the operation and organization of information, but rather helps stakeholders to make better decisions. Visuals have a more powerful effect on problem-solving performance and learning than text, and a combination of text and visuals is much more effective than text alone [23].

## 3. User Analysis

### 3.1. System Design

The system prototype was geared toward construction and civil engineering development projects. The system was designed to offer four simulations associated with a construction project: (1) hydrological water flow, (2) oil spills, (3) air quality due to microscopic or particulate matter, and (4) airflow. The water and oil simulations predicted the likelihood of floods before, during, and after the construction process, while the wind simulation was developed to visualize the airflow before and after construction. In addition, wind simulation has some application to be used in the context of air pollution. The algorithm process in this study did not compare quantitative measures with data in the field, but we believed that the methods should be quite similar and support in preparation of the EIA report.

The proposed system offers the advantages of openness and accessibility. Previous systems have mostly operated in stand-alone mode [4,24,25,26]. On the other hand, the proposed system is implemented as a pure web service that does not use non-standardized technologies such as Active X, applets, and Flash. Stand-alone and client server modes are commonly employed because simulations and 3D rendering require high computational power [26]. The proposed system attempts to overcome this limitation using a pure web service.

#### 3.1.1. Algorithms and Models

The simulations were designed using the cellular automata simulation method [27]. For the simulation of the hydrological water flow, interactive terrain modeling using hydraulic erosion was used. Interactive terrain modeling was built upon different physics-based erosion and deposition algorithms [28]. Each frame of a pixel represents a water column, which has the following information: (1) the layers of the material it consists of, (2) the depth of those layers, (3) the tilt angle, and (4) the height of the water.

Using pixel-level information, the model automatically calculates the water flow, the sediment change, and how it spreads across the terrain. The height of a water column represents the acceleration of the water flow. The height of the columns indicates the sum of the height of different terrain layers and water layers.

Based on the height differences between water columns, the acceleration, flow, and velocity of the water can be calculated, which can then be used to determine the changes in the sediment by pixel. By analyzing the interaction between the water columns and the layers, tilt angle, and regolith thickness, the algorithm predicts the interactions of the following water columns. The algorithm predicts the change across the entire terrain, not just the interactions between the water columns.

**Figure 2:**
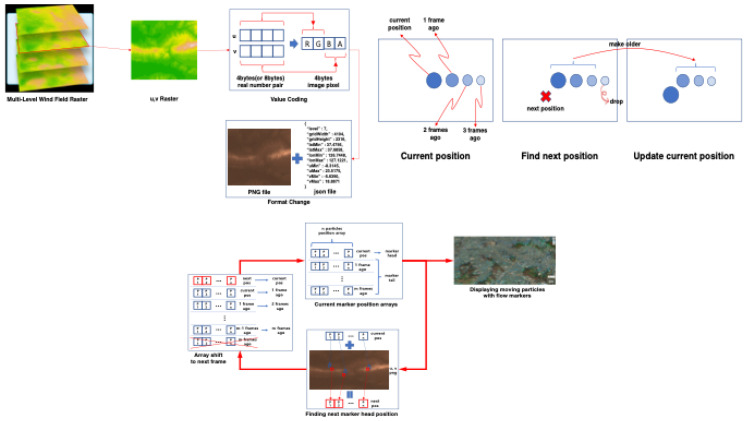
Raster data, the determination of wind flow markers, and the visualization of wind data.

For the wind flow, we visualized the results of the simulation data in raster format rather than the data itself (Figure 2). The streamline was calculated based on wind field data.

#### 3.1.2. Hydrological Water Flow System

**Figure 3:**
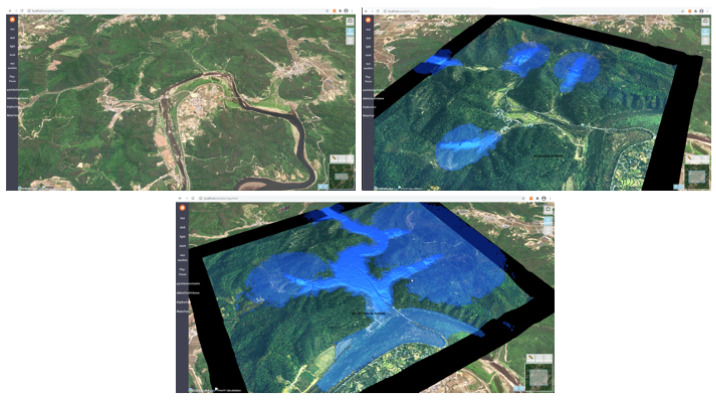
Hydrological water flow simulation.

The simulation presents the risk of flooding before and after a construction project (Figure 3). Given that the development area will change as the development progresses, the system presents the predicted changes to the river, particularly the damage occurring near the river due to flooding.

#### 3.1.3. Interactive Water Simulation

**Figure 4:**
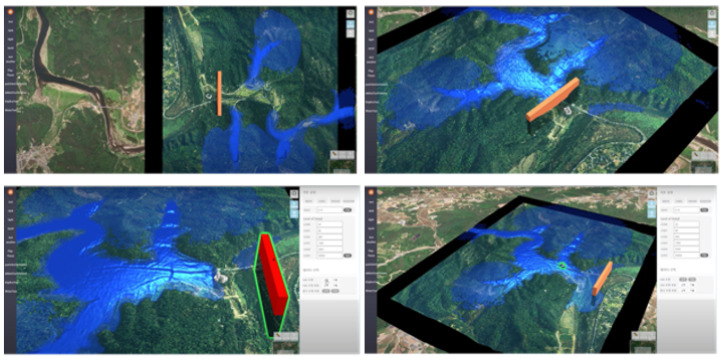
Interactive water simulation.

This simulation visualizes the hydrological water flow and simulates changes depending on user input, including embankment removal or construction (Figure 4). The orange and red shapes represent the height of the flood gate. In the interactive water simulation, users can freely adjust the placement and height of the flood gate. At the same time, they can set the area for water contaminant.

#### 3.1.4. Wind Simulation

**Figure 5:**
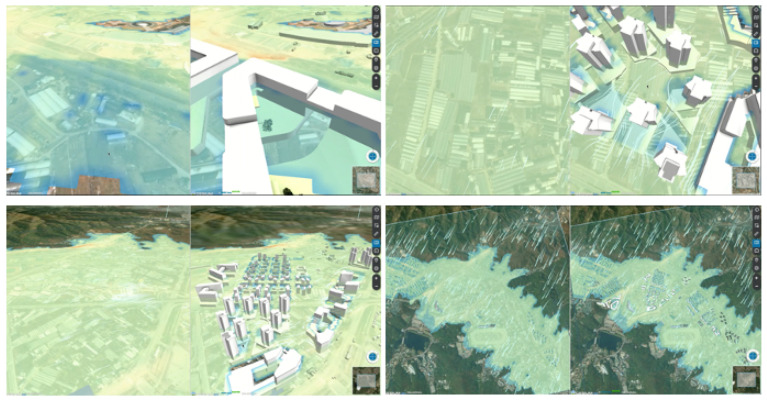
Wind simulation.

This simulation demonstrates the changes in the wind paths before and after the construction of a new town (Figure 5). When the town is constructed, the wind speeds increase. The white arrows represent the flow of the wind, showing both the direction and speed.

#### 3.1.5. Oil and Wastewater Simulation

**Figure 6:**
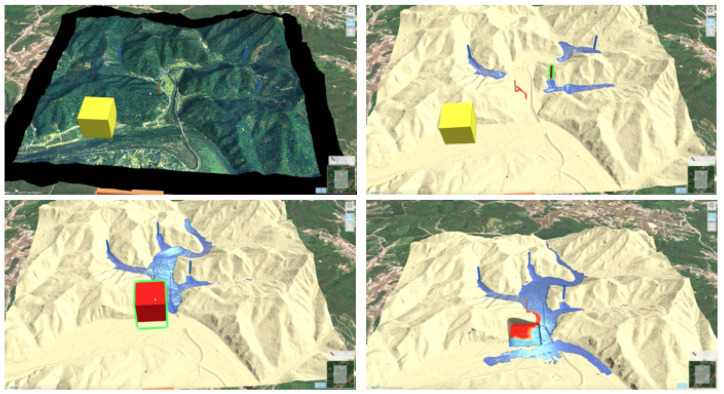
Flow of oil and wastewater based on the adjustment of geographical features.

This simulation visualizes the flow of oil and wastewater by adjusting the geographical features in real-time (Figure 6). The yellow elements represent a mix of wastewater and oil that has not spread into the environment, while the red elements represent the same pollutants that have been emitted into the surroundings. The blue area depicts the flow of oil and wastewater over the surface.

### 3.2. Participants and Measurements

We recruited 107 participants (55 female, 52 male) for this study online and on campus at Cornell University. We attempted to recruit a similar proportion of participants in each age group because we believed that age might be highly correlated with computer self-efficacy and perception of system usability. Thus, 27.41% of the participants were in their 20 s, 33.33% were in their 30 s, 9.63% were in their 40 s, and 29.63% were in their 50 s.

#### 3.2.1. Computer Self-Efficacy Survey

Computer self-efficacy is a self-rated measure of specific computer-related knowledge and skills [29]. In the present survey, the computer self-efficacy survey asked the participants to imagine that they are given a new software package for some aspect of work. The type of software package does not matter, but the package is designed to make a job easier. Participants rate their confidence in their ability to use the new software package from 1 to 10. The full survey is provided in Appendix A.

#### 3.2.2. System Usability Scale (SUS)

System usability surveys are considered a quick, reliable, and valid method for assessing the learnability and usability of a system [30]. Standardized questions are widely used to evaluate the usability of a system. An advantage of a system usability scale (SUS) is that it can be used with a diverse range of contexts, technologies, and systems. In addition, it demonstrates the general and overall quality of a system and its appropriateness for a specific context [30]. The SUS in the present study used a 5-point Likert scale [31] to assess the level of agreement with 10 statements, resulting in a score from 0 to 100 points, with higher scores demonstrating higher subjective usability. The full survey is provided in Appendix B.

### 3.3. Procedures

Participants first provided their demographic information, including gender, age, race, whether they were working in an environment-related field, and their computer self-efficacy [32]. They then explored the system, with the different simulations presented in random order. The participants used a MacBook Pro laptop with a mouse when exploring the system. The participants were given a brief explanation of each simulation. After the explanation, they were asked to click buttons and use the system without any instructions for each simulation. After five minutes of exploration, they answered the SUS [30].

## 4. Results

A linear mixed-model analysis was conducted to test the effects of the different simulations on the SUS scores. Several variables, such as gender, age, and environmental expertise, were included.

### 4.1. Impact of System Simulations on SUS Scores

The results showed that there were no significant differences between the four different simulations in the SUS scores (Table 2).

### 4.2. Impacts of Individual Differences on SUS Scores

#### 4.2.1. Environmental Expertise

The results showed that people working in the environmental field scored higher (+0.631) on the SUS than did those not working in the field, and this difference was statistically significant (*p* < 0.05).

#### 4.2.2. Computer Self-Efficacy

People who had higher computer self-efficacy had higher SUS scores than those with low self-efficacy (+0.102), and this difference was also statistically significant (*p* < 0.001)

#### 4.2.3. Interaction Effect

Computer self-efficacy showed a significant interaction effect with environmental expertise (Table 3). Among participants who had higher computer self-efficacy, those who were also environmental experts scored 0.173 higher on the SUS than participants who were not experts (*p* < 0.001).

## 5. Discussion

Since their introduction as part of the NEPA [3], EIAs have been instituted in more than 100 countries, serving not only as a major instrument for environmental management [33] but also as an effective tool for sustainable development that takes into account environmental, social, and economic factors. However, due to the complexity of EIAs in terms of the elements they are expected to cover (Table 1), stakeholders often find it difficult to understand EIA reports, thus, they cannot participate effectively in the establishment of development plans and related decisions.

Furthermore, SEA aims to integrate environmental considerations into plan-making, and so to promote sustainable development and environmental protection. Global visualization of EIA and SEA processes generally entails the preparation of an environmental report that identifies the likely significant impacts of the levels in a plan/programme (SEA) as well as projects (EIA), proposal of measures to avoid or mitigate any significant negative impacts, consultation on the report with the public and government environmental bodies, taking the environmental report and consultation findings, and monitoring of the actual impacts of the plan/programme/projects helping with visualization [7].

In particular, for a potential construction project, local residents are a large stakeholder group, but they are typically not organized and have a lower social status and limited financial resources. Therefore, though EIAs play a positive role in the public decision-making process [34], meaningful public participation in the EIA process is lacking, leading to an imbalance between relevant stakeholders [35].

The importance of involving the public in the design process for visual tools has been clearly outlined by the International Association for Public Participation (IAP2), who identify it as a core value of public participation: “public participation seeks input from participants in designing how they participate” [36,37]. This is particularly important because effective images evoke powerful emotions, driving decisions and actions. This key feature of visual tools can be used to support an effective, meaningful, and participatory dialogue, contributing to enhanced knowledge brokerage [38]. The main purpose of the DSS proposed in the present study for EIAs was thus to help stakeholders communicate more effectively and enhance social sustainability. The results showed that respondents with a higher computer self-efficacy tended to rate the system as more usable than those who had lower computer self-efficacy. This suggests that engineers and environment administrative officers should benefit from the DDS in the decision-making process because they are assumed to have higher computer self-efficacy [34].

Visualization has also been advocated as an instrument of change towards more sustainable patterns of behavior and development [39] and as a tool to support the achievement of Sustainable Development Goals.

In addition, environmental experts were more satisfied with this system than were members of the general public with no environmental expertise. This indicates that the DDS could be further improved by servicing the general public who have no environmental background. However, this system could be employed in other countries with other stakeholders because there are no barriers in terms of language or cultural/policy differences. In addition, it could be useful in the context of public hearings.

## 6. Limitations and Future Studies

This study has some limitations. First, the system was designed in a 2D format that could be extended to a 3D form in the future. Thus, to design a system that helps the general public better understand the EIA process, future studies could apply 3D modeling techniques. Second, even though EIAs are employed in a variety of countries, differences exist between them. Third, a system that visualizes other elements that are not covered by the six types of environment and their associated items in a typical EIA is needed (Table 1). Our study shows that this system would help to increase environmental literacy. Moreover, in this study we only demonstrated the issues such as waterflow, air movement, and oil spill as examples, but in the future all of the 21 items (Table 1) will be implemented to help the decision-making process; therefore, items like landscape, noise/vibration, and sunlight should be further studied.

## 7. Conclusions

In this paper, we designed and presented a DSS that assists decision-making in the EIA process for a variety of stakeholders. The system visualizes for the user the environmental conditions before and after a construction project. To better understand the perceived usability for different stakeholders, we explored the impact of individual differences, including computer self-efficacy and environmental expertise, on SUS scores. The results obtained from 107 participants exploring the system and answering a survey showed that the participants with higher computer self-efficacy and environmental experience scored more highly on the SUS. We expect that our proposed system could help improve the communication between the public and experts during public hearings.

## Figures and Tables

**Table 1:** Six environments and associated items covered by an EIA in South Korea (Environmental Impact Assessment Act, 2018).

Environment (No. of Items)	Items
Atmospheric (4)	Weather, air quality, odors, greenhouse gas emissions
Soil (3)	Land use, soil, topographic/geological features
Water (3)	Water quality (ground and underground), hydraulics/hydrology, marine environment
Living (6)	Environmentally friendly resource circulation, noise/vibrations, recreation/landscape, hygiene and public health, radio interference, barriers to daylight
Bio-ecological (2)	Plants and animals (land and ocean), natural environmental assets
Socio-economic (3)	Population, housing, and industry

**Table 2:** Mean SUS scores for the four simulations on SUS (standard deviation in parentheses).

Dependent Variable	Hydrological Water Flow	Interactive Water	Wind	Oil and Wastewater
SUS Score	3.43 (1.47)	3.37 (1.46)	3.49 (1.41)	3.33 (1.52)

**Table 3:** Results of linear mixed models for computer self-efficacy and environmental expertise.

Factor	Estimate	Standard Error	DFDen	T Ratio	Prob > |*t*|
Intercept	2.924	0.344	243.1	8.50	<0.0001 *
Computer Self-Efficacy	0.102	0.039	307	2.61	0.0095 **
Environmental Expertise	0.631	0.074	305.9	8.50	<0.0001 ***
Computer Self-Efficacy * Environmental Expertise	0.173	0.039	307.9	4.48	<0.0001 ***

* *p* < 0.05, ** *p* < 0.01, *** *p* < 0.001.

## Appendix A. Computer Self-Efficiency Survey

I could complete the job using the software package if…

1. There was no one around to tell me what to do as I go.
2. I had never used a package like it before.
3. I had only the software manuals for reference.
4. I had seen someone else using it before trying it myself.
5. I could call someone for help if I got stuck.
6. Someone else had helped me get started.
7. I had a lot of time to complete the job for which the software was provided.
8. I had just the built-in help facility for assistance.
9. Someone showed me how to do it first.
10. I had used similar packages before this one to do the same job.

## Appendix B. System Usability Scale Survey Items

1. I think that I would like to use this system frequently.
2. I found the system unnecessarily complex.
3. I thought the system was easy to use.
4. I think that I would need the support of a technical person to be able to use this system.
5. I found the various functions in this system were well integrated.
6. I thought there was too much inconsistency in this system.
7. I would imagine that most people would learn to use this system very quickly.
8. I found the system very cumbersome to use.
9. I felt very confident using the system.
10. I needed to learn a lot of things before I could get going with this system.
